# Mast Cell Activation Syndrome in COVID-19 and Female Reproductive Function: Theoretical Background vs. Accumulating Clinical Evidence

**DOI:** 10.1155/2022/9534163

**Published:** 2022-06-22

**Authors:** Dariusz Szukiewicz, Piotr Wojdasiewicz, Mateusz Watroba, Grzegorz Szewczyk

**Affiliations:** Department of Biophysics Physiology & Pathophysiology, Faculty of Health Sciences, Medical University of Warsaw, Warsaw, Poland

## Abstract

Coronavirus disease 2019 (COVID-19), a pandemic disease caused by severe acute respiratory syndrome coronavirus 2 (SARS-CoV-2) infection, can affect almost all systems and organs of the human body, including those responsible for reproductive function in women. The multisystem inflammatory response in COVID-19 shows many analogies with mast cell activation syndrome (MCAS), and MCAS may be an important component in the course of COVID-19. Of note, the female sex hormones estradiol (E_2_) and progesterone (P4) significantly influence mast cell (MC) behavior. This review presents the importance of MCs and the mediators from their granules in the female reproductive system, including pregnancy, and discusses the mechanism of potential disorders related to MCAS. Then, the available data on COVID-19 in the context of hormonal disorders, the course of endometriosis, female fertility, and the course of pregnancy were compiled to verify intuitively predicted threats. Surprisingly, although COVID-19 hyperinflammation and post-COVID-19 illness may be rooted in MCAS, the available clinical data do not provide grounds for treating this mechanism as significantly increasing the risk of abnormal female reproductive function, including pregnancy. Further studies in the context of post COVID-19 condition (long COVID), where inflammation and a procoagulative state resemble many aspects of MCAS, are needed.

## 1. Short Introduction: Signaling the Problem

COVID-19, a pandemic disease caused by severe acute respiratory syndrome coronavirus 2 (SARS-CoV-2) infection, can affect virtually all systems and organs of the human body, including those responsible for reproductive function in women [1]. Considering COVID-19 as a multisystem inflammatory response, the disease may reveal close analogy with conditions related to upregulation of the secretory activity of mast cells (MCs) with increased susceptibility to degranulation or mast cell activation syndrome (MCAS) [2]. The relationship between COVID-19 and reproductive function in women becomes clearer after taking into account the fact that female sex hormones, including estradiol (E_2_) and progesterone (P4), significantly influence mast cell (MC) behavior [3].

## 2. MCs and MC-Derived Mediators in a Woman's Reproductive System and Placenta

MCs are very special cells of the immune system derived from pluripotent cells of the myeloid lineage and are widely distributed in connective tissues throughout the body, including the reproductive system and placental tissue of women [4–6]. Unlike other white blood cells, MCs present in blood are immature in the form of MC progenitors generated from CD34^+^ hematopoietic stem cells, and these cells become fully competent after recruitment into the tissue at target localization [7]. Terminal differentiation of MCs occurs under the influence of c-kit ligand (CD117) and cytokine stem cell factor (SCF) in the local site microenvironment containing other distinct cytokines (e.g., IL-3 and IL-33), especially growth factors [8]. Thus, heterogeneity of the population of MCs is decisively observed because they reach their full maturity at the target site. This heterogeneity includes significant differences in the cell ultrastructure, morphology, mediator content, and surface receptors [9]. For example, based on the secretory granule content, human MCs are described as either MC_TC_, which contains two serine proteases (tryptase and chymase) together with carboxypeptidase and cathepsin G, or MC_T_, which contain only tryptase [10, 11]. The release of key mediators during the activation and degranulation of MCs, including COVID-19-related MC activation, may significantly affect the course of many physiological and pathological reproductive processes in the human body, primarily influencing the permeability of vessels and vascular tone and indirectly modulating electrolyte and water balance or contents of the extracellular matrix [12, 13]. Interestingly, the majority of the key physiological processes in the female reproductive tract, including menstruation, follicle development, ovulation, implantation, pregnancy, labor, and postpartum remodeling, consist of a readily visible inflammatory background [14]. Research successively provides new data. At present, the demonstrated roles of MCs and MC-derived mediators in the human female reproductive system in health and disease are summarized in Table 1.

It is worth noting the presence of MCs in neuroendocrine organs within the female reproductive system, such as ovaries and uterus, where the role of MCs still requires a better understanding [83]. Moreover, the functional status of MCs may be modulated by the female sex hormones E2 and progesterone P4 [3]. For example, NLRP3 inflammasome activation of MCs by estrogen via the nuclear-initiated signaling pathway contributes to the immune inflammatory response in endometriosis and further spreading of the disease [84]. MCs express E2 (ER*α*, ER*β*, and G protein-coupled estrogen receptor (GPER) and P4 receptors (PR-A and PR-B) and further respond to these hormones, which causes changes in MC cell number, distribution, and degranulation in ovarian tissue [3, 85]. E2 and P4 upregulate chemokine receptor expression on MCs and promote their migration into the fetal-maternal interface with subsequent increased production of MC mediators. During the process of oocyte ovulation, E2-induced MC degranulation seems to be a crucial releasing factor, whereas P4 action is required in the peri-implantation period to initiate uterine spiral artery remodeling by decidual MCs [67, 86–88].

## 3. Secretory Activity of MCs: Main Categories of the Mediators, the Mechanisms of Activation and Degranulation

MCs together with adjacent dendritic cells act as sentinels for tissue damage and pathogen invasion by the release of various mediators from different compartments following diverse stimuli [89]. However, the interactions of MCs with viruses and viral products can have detrimental consequences, leading to a cytokine storm, as described in SARS-CoV-2 infection [90].

Contrary to the popular belief that MC degranulation is nonspecific based on an all-or-none law process in which many mediators are released, it is now apparent that specific mediators are secreted in response to particular stimuli or pathologic states [91].

MC mediators can be divided into three overlapping categories: preformed mediators, newly synthesized lipid mediators, and cytokines, including chemokines (Table 2).

MC secretory granules or secretory lysosomes reveal heterogeneity, and their heterogenous nature reflects many factors, including the tissue of residence and the species, health status and, to some degree, age of the individual [117, 118]. Following MC activation, preformed mediators are rapidly (within seconds to minutes) released into the extracellular environment. Degranulation with or without *de novo* synthesis of the mediator dominates the secretory profile of MCs; however, other options are also described therein, including *de novo* synthesized mediator release without degranulation [117] Table 2.

The typical and best-known mode of MC activation with the release of their granule content is associated with allergic reactions mediated via E class immunoglobulin (IgE) binding to the high-affinity receptor for immunoglobulin E (Fc*ε*RI) expressed on the MC surface [119]. Tetrameric Fc*ε*RI consists of the IgE-binding *α*-chain, the multimembrane spanning the *β*-chain, and a disulfide-linked homodimer of the *γ*-subunit (Fc*ε*R*γ*). The signaling pathway induced after Fc*ε*RI binds with IgE includes the activation of Lyn, one of several Src-family tyrosine kinases in immune cells, which phosphorylates tyrosines in its immunoreceptor tyrosine base activation motifs (ITAMs) on the *β* and *γ* chains of Fc*ϵ*RI. Next, Lyn induces Syk tyrosine kinases to phosphorylate the signaling proteins that serve as the linkers for T cell activation (e.g., LAT1 and LAT2) [120]. Hydrolysis of phosphatidylinositol-4,5-bisphosphate leads to the formation of inositol-1,4,5-triphosphate (IP3) and diacylglycerol (DAG), the second messengers for mobilizing intracellular calcium from the endoplasmic reticulum [121]. The influx of calcium ions produces both activation and translocation (toward the nucleus of the cell) of NF-*κ*B with subsequent transcription of genes encoding various cytokines, including IL-1, IL-2, IL-6, IL-8, IL-12, IL-13, and TNF*α* [122]. The zinc finger transcription factor Zeb2 (also named Sip1, Zfhx1b) regulates both early and late MC responses related to Fc*ε*RI-mediated degranulation [123]. Complementary to the Lyn signaling pathway, another member of the Src family of tyrosine kinases, Fyn, is involved in the regulation of MC degranulation upon Fc*ϵ*RI activation. Fyn is involved in the phosphatidylinositol-4,5-bisphosphate 3-kinase (PI3K)/protein kinase B (Akt)/mammalian target of rapamycin (mTOR) signaling pathway, which ultimately leads to cytokine production and MC chemotaxis [124].

MCs also express Fc*γ*R, a receptor for IgG. The signal from Fc*γ*R can crosstalk with Fc*ϵ*RI because the *γ*-chain homodimer is identical in Fc*γ*RI and Fc*ϵ*RI [119]. In addition to the mentioned Fc*ϵ*RI and Fc*γ*R, MCs express other receptors involved in their activation and/or immune response. Some of the most important receptors include Fc receptors for IgA and IgG; a G protein-coupled receptor known as MRGPRX2; angiotensin-converting enzyme 2 (ACE2) receptor; Toll-like receptors (TLRs) and other receptors for pathogen-associated molecular patterns (PAMPs); receptors for cytokines/chemokines, corticotropin releasing hormone, estrogen and progesterone; and complement receptors (e.g., C3a protein) [125–127]. Evidence indicates that the spike protein (S protein) is the main viral structure that SARS-CoV-2 uses to fuse with and enter human host cells by binding to the ACE2 receptor. This binding is followed by activation of the enzyme furin. Furin plays a vital role for the virus because the spike protein of SARS-CoV-2 must be cleaved by furin or furin-like proteases to become fully functional [128, 129]. Despite the fact that ACE2 is widely expressed in many different cells, including MCs, and is the one major receptor for SARS-CoV-2, other host receptors and/or coreceptors that promote the entry of the virus into cells have been reported recently [129].

**Figure 1:**
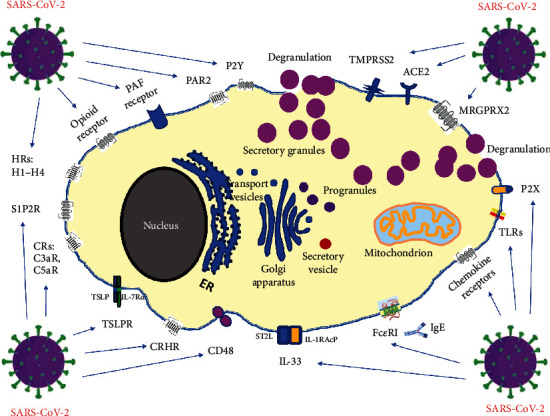
Receptors with a documented role in MC activation and degranulation in the context of possible triggering by coronavirus/COVID-19.

Receptors with a documented role in MC activation and degranulation in the context of possible triggering by coronavirus/COVID-19 are shown in Figure 1.

- The best-known mode of MC activation with degranulation and subsequent allergic reactions is mediated via IgE binding to Fc*ε*RI expressed on the MC surface. MCs also express Fc*γ*R (not marked in the figure), a receptor for IgG. The signal from Fc*γ*R can crosstalk with Fc*ϵ*RI because the *γ*-chain homodimer is identical in Fc*γ*RI and Fc*ϵ*RI [119]
- The spike protein (S protein) is the main viral structure that SARS-CoV-2 uses to fuse with and enter human host cells by binding to ACE2 [128, 130]. MCs express the renin-angiotensin system, the ectoprotease ACE2 required for SARS-CoV-2 binding, and serine proteases, including TMPRSS2, required for priming of the corona spike protein [130]. Such triggers could lead to secretion of multiple proinflammatory mediators selectively, without release of histamine or tryptase [131]
- IL-33 expression in response to SARS-CoV-2 correlates with seropositivity in COVID-19 convalescent individuals [132]. IL-33 needs the specific receptor ST2L and IL-1RAcP heterodimer for its binding, which stimulates the production of different types of cytokines and chemokines that have crucial roles in the exacerbation of allergic diseases and inflammation. Moreover, IL-33 augments IgE-mediated MC activation and potently enhances the human MC reactivity to C3a and C5a (degranulation, cytokine, and chemokine release), independent of changes in C3aR or C5aR receptor expression or the level of Ca2+ influx [133]
- Elevated CRH level in COVID-19 may activate CRHR on MCs influencing vascular permeability. It was demonstrated that even COVID-19-related psychological stress is capable of inducing MC-related diseases, including mastocytosis. Psychological stress, by stimulating the release of corticotrophin releasing hormone (CRH) into the serum, can induce MC degranulation [134]
- Circulating S1P emerged as negative biomarker of severity/mortality of COVID-19 patients [135]. S1P generation induces calcium mobilization leading to MC degranulation. S1P affects immune cell function mostly by acting through its receptor S1P2R at the cell membrane but it can also induce S1P receptor-independent responses in the cells where it is generated
- Development of clinical COVID-19 involves dysfunctional MC activation and histamine release. It was suggested that histamine signaling pathway may be a potential therapeutic target to prevent COVID-19 from progressing to acute respiratory distress syndrome (ARDS) [136]. All known histamine receptors are expressed on MCs, but H1 and H4 have important roles in the progression and modulation of histamine-mediated allergic/inflammatory diseases
- Human MCs possess opioid receptors, which when stimulated trigger degranulation. The opioid system consists of four types of receptors: mu (M*μ*), delta (*δ*), kappa (*κ*), and opioid receptor like-1 (ORL1) [137]. Opioid receptors may also be capable of modulating IgE-mediated histamine release [138]
- TSLPR signaling is required for MC development and aggravates allergic reactions through the activation of mouse double minute 2 homolog (MDM2) and signal transducer and activator of transcription 6 (STAT6). TSLPR is formed by the signaling complex: TSLP and IL-7Rɑ [139]
- PAF is generated by cells involved in host defense in a variety of inflammatory conditions in which MCs accumulate. PAF is capable of activating MCs and inducing a chemotactic response. COVID-19 manifestations, including pulmonary microthrombosis and inflammation, are mediated via PAF receptor [140]
- MRGPRX2 is a novel, low-affinity and low-selectivity receptor, to activate MCs in an IgE-independent manner [141]. This receptor has been cited as a therapeutic target for COVID-19 [142]
- MCs respond to TLR ligands by secreting cytokines, chemokines, and lipid mediators, and some studies have found that TLR ligands can also cause degranulation, although this finding is contentious [143]
- Several clinical investigations revealed that chemokines are directly involved in the different stages of SARS-CoV-2 infection [144]. At least nine chemokine receptors (CXCR1, CXCR2, CXCR3, CXCR4, CX3CR1, CCR1, CCR3, CCR4, and CCR5) have been described to be expressed by human MCs of different origins [145]
- Tryptase released from MCs can activate PAR-2 in an autocrine or paracrine manner [146]. It was suggested that PAR by proteases plays a role in COVID-19-induced hyperinflammation [147–149]
- Purinergic P2X ionotropic nucleotide receptors are ligand-gated ion channels activated by extracellular ATP and selective for monovalent and divalent cations (Na+, K+, and Ca2+), whereas purinergic G protein-coupled receptors P2Y are stimulated by nucleotides such as adenosine triphosphate, adenosine diphosphate, uridine triphosphate, uridine diphosphate, and UDP-glucose [150, 151]. Purinergic signaling is involved in the pathophysiology of several viral infections which makes the purinergic system a potential target of investigation in COVID-19 [152]
- CD48 is in the CD2 subfamily of the immunoglobulin (Ig) superfamily and shares many structural features with other Ig family members. CD48 expression increases under inflammatory conditions [153]

Abbreviations in Figure 1 are as follows: ACE2: angiotensin-converting enzyme 2 receptor; CD48: cluster of differentiation 48 also known as B-lymphocyte activation marker (BLAST-1); CRHR: corticotropin-releasing hormone receptor; CRs: C3aR, C5aR—complement receptors; FcεRI: high-affinity receptor for the Fc region of immunoglobulin E (IgE); GPER: G protein-coupled estrogen receptor; HRs: H1-H4—histamine receptors; IgE: E class immunoglobulin; IL-1RAcP: IL-1 receptor accessory protein; IL-33: interleukin-33; IL-7Rɑ: IL-7 receptor ɑ chain; ST2L: suppression of tumorigenicity 2, IL-33 receptor; MRGPRX2: Mas-related G-protein-coupled receptor X2; P2X: ligand-gated ion channels (ionotropic receptors); P2Y: metabotropic purinergic receptors; PAF receptor: platelet-activating factor receptor; PAR2: protease-activated receptor 2; S1P2R: sphingosine-1-phosphate receptor 2; TLRs: Toll-like receptors; TMPRSS2: transmembrane serine protease 2; TSLP: thymic stromal lymphopoietin; TSLPR: thymic stromal lymphopoietin receptor.

Participating in intracellular signaling and promoting the production of proinflammatory cytokines involved in the regulation of the innate immune response, reactive oxygen species (ROS) stimulate MC degranulation via high-affinity receptors to IgE (Fc*ε*RI) [154]. Although ROS are intermediaries released during normal oxygen metabolism, deleterious effects of oxidative stress associated with an overabundance of ROS are also well documented with regard to female reproductive tissues in the context of an inflammatory background of infertility [155]. In healthy individuals, ROS and antioxidants are maintained in a balanced state, and the antioxidant systems in the ovary and endometrium are sufficient to limit the production of ROS, inactivate them, and repair cell damage [156, 157].

A local increase in the concentration of MC mediators in surroundings containing other MCs upregulates the secretory activity of these MCs, including susceptibility to degranulation. Such an activation syndrome may occur with an unchanged number of MCs as nonclonal MC activation syndrome (nc-MCAS) or together with MC hyperplasia in the form of MC activation syndrome (MCAS) [158–160]. Thus, changes in hormone and cytokine receptor expression caused by MC hyperplasia may be important when comparing MC activation in MCAS vs. nc-MCAS [160]. As proteases, mast cell tryptases are the only currently available convenient marker of human mast cell activation and burden [161]. Of note, tryptases are the most abundant proteases of the human MCs, comprising up to 20% of cellular protein [162]. Nevertheless, the major component of the granules that represents at least 30% of their dry weight is heparin, which is synthesized by MCs as a proteoglycan [109].

Notably, epigenetic mechanisms are also involved in MC activation and proliferation [163]. Numerous microRNAs (miRNAs) or small single-stranded noncoding RNA molecules containing 18 to 25 nucleotides that function in RNA silencing and posttranscriptional regulation of gene expression have been examined in the context of MC biology [164]. Acting via Fc*ϵ*RI alone or together with increased levels of interleukins (IL-33, IL-10), the respective mRNAs may trigger signals for increased MC degranulation (miR-142-3p, miR-126), PI3K/Akt signaling pathway-dependent increased Ca^2+^ influx with degranulation (miR-155), or reduced MC migration with proliferation and increased degranulation (miR-221/222) [127, 165–167].

## 4. COVID-19-Related MCAS or nc-MCAS and Possible Consequences for Female Reproductive Function

**Figure 2:**
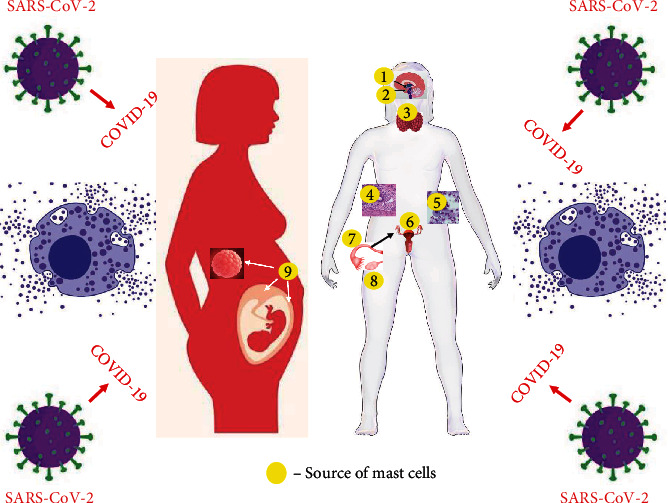
The potential sites of mast cell activation syndrome (MCAS) that may affect female reproductive function. There are indications to believe that SARS-CoV-2 infection and related COVID-19 may produce release of MC mediators from MC sources within female reproductive organs and other related to reproduction organs, such as 1: hypothalamus; 2: pituitary gland; 3: thyroid gland; 4: endometrial tissue outside the uterus (endometriotic foci); 5: peritoneal fluid; 6: uterus (endometrium, myometrium), 7: fallopian tube; 8: ovary; and 9: blastocyst, placenta, and decidua. Surprisingly, in majority of cases, histologic rationale for increased risk of MCAS (i.e., presence of mast cells in the respective organs) is not confirmed by pathophysiology (i.e., clearly proven relationship: MCAS⟶reproductive disorder), and so far, clinical data do not support that COVID-19 triggers MCAS in female reproductive system. See the main text (Section 4) for details.

As a pandemic disease, COVID-19 raises interest in many aspects of female reproductive health effects [1, 168]. Knowledge on this subject is constantly being generated, and there is evidence that MC activation together with the induction of a cytokine storm may play a vital role in the pathomechanism of some reproductive disorders accompanying COVID-19 [2, 11]. The potential sites of these disorders are pointed in Figure 2.

### 4.1. Hormonal Disorders

Hormonal disorders during COVID-19 vary, just as the response to SARS-CoV-2 infection varies [169, 170]. The changes in hormonal activities concern both the hormones directly regulating the menstrual cycle (luteinizing hormone (LH), follicle-stimulating hormone (FSH), and the female sex hormones E_2_ and P4) and the hormones that may indirectly modulate its course (e.g., prolactin, thyroid-stimulating hormone (TSH), androgens, and glucocorticoids) [169, 171]. It was reported that approximately one-fifth of COVID-19 patients exhibited a menstrual volume decrease or cycle prolongation, approximately one-fifth exhibited menorrhagia, and approximately 30% experienced new dysmenorrhea [172, 173]. Considering the inflammatory background of menstruation, these last two symptoms and the worsening of already existing heavy menstrual bleeding or/and premenstrual symptoms may indicate the involvement of MC activation in the pathogenesis of cytokine storms in COVID-19 [2, 173]. Since P4, androgens, and glucocorticoids reveal natural immunosuppressive properties, it should be realized that E_2_ is implicated in the immune response as an enhancer, including MC activation [174–177].

Among the estrogen receptors, GPER is responsible for the various running fast nongenomic effects of estrogens, including degranulation of MCs [178].

Unlike after activation of genomic pathways by classical nuclear ER*α* and ER*β* receptor stimulation, nongenomic cellular responses to estrogen are initiated at the plasma membrane and result in rapid activation of second messenger-triggered cytoplasmic signal transduction cascades. Ultimately, after E2 binds to GPER, it mobilizes intracellular calcium and synthesizes phosphatidylinositol (3,4,5)-trisphosphate (PIP3) in the nucleus [179]. After estrogen binding, GPER may functionally cross-react with diverse cell signaling systems, such as the epidermal growth factor receptor (EGFR) pathway, the Notch signaling pathway, and the mitogen-activated protein kinases (MAPK) pathway [180].

Paradoxically, despite the risk of cytokine storms following MC degranulation, the net effect of E_2_-related modulation of immune cells may be beneficial in SARS-CoV-2 infection. Based on preliminary research results, women with high levels of E_2_ exhibited a lower risk of developing severe COVID-19 symptoms and a lower incidence of death [181]. On the other hand, serum E_2_ levels in patients with COVID-19 may reflect the severity of infection, as any acute critical illness can lead to suppression of the hypothalamic–pituitary–ovarian axis, biochemically manifesting as low FSH, LH and E_2_ [182]. Thus, the mechanisms of E_2_ action in viral infections, including COVID-19, should be further comprehensively studied to provide a rationale for therapeutic strategies.

Thyroid hormones are vital for the proper functioning of the female reproductive system given that they modulate the metabolism and development of ovarian, uterine, and placental tissues. Hyperthyroidism is common in female patients with COVID-19 and coexists with thyroid-stimulating hormone (TSH) suppression that seems to be related to increased levels of the proinflammatory cytokine IL-6 [183, 184]. Based on recent studies, activation of MCs with a significantly higher number of degranulated MCs and histamine-induced overexpression of glycoprotein CD86 on antigen-presenting cells within thyroid gland tissue may be responsible for hyperthyroidism [185]. Moreover, MCs can synthesize and store TSH and the thyroid hormone triiodothyronine (T3) [186]. More research is needed to determine whether MCAS-related hyperthyroidism in COVID-19 has a significant impact on reproductive function in women.

### 4.2. Women with Endometriosis

Endometriosis is an estrogen-dependent and progesterone- (P4-) resistant inflammatory disorder of unknown etiology that affects 5–10% of women of reproductive age and is characterized by the presence of endometrial tissue outside the uterine cavity [187]. Patients with endometriotic foci (lesions) constitute up to 80% of women with pelvic pain and 20-50% of women with infertility. MCs are very prevalent in endometriosis tissue, and many MCs appear to be activated and degranulated. MCs and their products, tryptase, histamine, and many other proinflammatory cytokines, could contribute significantly to several features of endometriosis [76]. It was proposed that MCs represent a therapeutic target in endometriosis to assure better control of endometriosis inhibition and symptom relief [82]. Not surprisingly, endometriosis is included in the list of symptoms and findings in MCAS [76, 84]. Despite the existence of undoubted dysfunction of the immune system in endometriosis, no evidence is available that shows that those with endometriosis are at an increased risk of contracting COVID-19. It is likely that endometriosis does not increase the susceptibility to COVID-19 infections but alters the manifestation of the disease [188]. The prevalence of the disease may depend on the interaction between the virus and the individual's immune system, but further studies are required in this regard. However, although not confirmed, women with thoracic endometriosis (in the lungs and/or diaphragm) may be at increased risk [189]. The manifestation of COVID-19 may be altered in endometriosis with a slightly decreased frequency of asymptomatic infection and fever and an increased frequency of rare symptoms (i.e., sore throat, nasal congestion, cough, shortness of breath, headache, weakness, and muscle pain, reduced sense of smell and/or taste, and ocular problems) [190]. Further studies involving a larger group of patients are needed. On the other hand, exacerbations in pelvic pain may be experienced due to high levels of inflammatory/pain mediators as well as pauses in medical or allied health treatment or postponement of surgical treatments. Evidence that COVID-19 by itself accelerates the progression/development of endometriosis is not available [190].

### 4.3. Female Fertility and the Course of Pregnancy

Despite the fact that fertility (including low sperm count, low sperm quality, and reduced sperm mobility) and sexual function may be disrupted in a portion of male patients as a result of SARS-CoV-2 infection [191, 192], there is no evidence that a history of asymptomatic or mild SARS-CoV-2 infection in females may negatively affect normal female fertility or affect embryo laboratory outcomes or clinical outcomes in assisted reproductive technology (ART) treatments [168, 172, 193, 194]. Moreover, to date, cohort studies of SARS-CoV-2-positive mothers, including both asymptomatic mothers and those with mild symptoms, did not reveal adverse effects on the mothers or neonates regardless of the timing of the infection (i.e., first, second, or third trimester) [194–196]. Naturally, in severe COVID-19 among pregnant women requiring critical care, higher complication rates should be expected, and these increased rates are particularly related to a higher incidence of iatrogenic prematurity due to induction of preterm delivery in the face of the threat to the mother's life [197].

This finding is puzzling considering that genes encoding the SARS-CoV-2 angiotensin-converting enzyme 2 (ACE2) receptor, which is required for virus entry, and transmembrane serine protease 2 (TMPRSS2), which is required for spike (S) protein priming, are coexpressed in the trophoblast of the blastocyst and the syncytiotrophoblast and hypoblast of the implantation stages, which develop into tissues that interact with the maternal blood supply for nutrient exchange [198, 199]. This finding may demonstrate a high risk of vertical transmission among SARS-CoV-2 infections (see later in this section). However, as data continue to accumulate, the final findings may depend on the number of SARS-CoV-2-positive patients tested. To date, there are no comprehensive reviews or completed meta-analyses that explore the association between COVID-19 and female fertility, especially in the context of MC activation. The strength of such activation should be proportional to the strength of ACE2 and TMPRSS2 coexpression.

Ongoing studies are focused on demonstrating the adverse effects of SARS-CoV-2 infection on ovarian reserve function (mean AMH decline, elevation of basal FSH or LH, and abnormal FSH/LH ratio), changed uterine receptivity due to affected endometrial thickness and/or morphology, disturbed subendometrial blood flow and/or uterine spiral artery blood flow, and altered fallopian tube function and menstrual status [1].

It is crucial to identify cells simultaneously expressing ACE2 and TMPRSS2. Based on the latest results, it can be assumed that the female reproductive tract has a low concentration of SARS-CoV-2 receptors or, more precisely, ACE2 and TMPRSS2 are coexpressed on the same cells [200]. These results suggest that the myometrium, uterus, ovaries, and fallopian tube are likely not susceptible to infection by SARS-CoV-2. In another study on preconceptional human endometrium, analysis of cell entry factors for SARS-CoV-2 (including ACE2 and TMPRSS2) by single-cell RNA-sequencing (scRNAseq) revealed a low risk of infection [201]. In contrast to the mentioned papers, coexpression of the SARS-CoV-2 entry molecules ACE2 and TMPRSS2 has been confirmed within the human ovarian cortex and medulla, including oocytes of different stages and granulosa cells [202]. Regarding the latter two locations, no marked difference in ACE2 or TMPRSS2 expression was observed between young and old ovaries and ovaries with low and high reserves [202]. Thus, SARS-CoV-2 may potentially target specific ovarian cells and affect ovarian function. Moreover, recent research suggests the possibility of noncanonical cell entry machinery for SARS-CoV-2 based on the new putative receptors/co-factors identified [201]. For example, in vitro studies have demonstrated interactions between the spike protein of SARS-CoV-2 and the innate immune system, including C-lectin type receptors (CLR), Toll-like receptors (TLR) and neuropilin-1 (NRP1), and the nonimmune receptor glucose-regulated protein 78 (GRP78), which are constantly expression in female reproductive tissues [203]. At the level of the endometrium across the menstrual cycle, alternative potential cell entry machinery components include BSG, ANPEP, CD209, CLEC4G, TMPRSS4, TMPRSS11A, FURIN, CTSB, CTSL, and IFITM1 [200]. All of the abovementioned receptors/cofactors can also be important in the context of the further mutagenic activity of SARS-CoV-2.

There are many unknowns regarding the vertical transmission of SARS-CoV-2 through the placenta. In general, hematogenous spread as a mechanism of infection and transmission across the placenta into the fetal circulation appears unlikely due to inefficient viral replication in placental tissues. The majority of newborns delivered from SARS-CoV-2-positive mothers test negative following delivery, suggesting that protective mechanisms exist within the placenta [204]. Placental tissues showed significantly lower expression of both ACE2 and TMPRSS2 compared with adult lung tissues. Placental samples expressed low to no ACE2, whereas TMPRSS2 seemed to be present at variable levels in the chorionic villi [205]. The possibilities that a time window exists during pregnancy during which ACE2 and TMPRSS2 are coexpressed and that individual variability may influence the strength of this coexpression cannot be excluded. One possible reason for discordant results within the published literature may be the widespread presence of Fc receptors in the human placenta, as these are known to bind antibodies to varying affinities and may therefore lead to nonspecific staining [206, 207].

It was discovered that placental cytotrophoblasts and syncytiotrophoblasts express ACE2 from 7 weeks onward, suggesting that SARS-CoV-2 could cross into the placenta at any gestational age [208]. Studies of ACE2 and TMPRSS2 coexpression in the developing embryo up to day 14 (from surplus IVF human embryos) revealed the colocalization of these genes, raising concern about increased susceptibility to SARS-CoV-2 fetal infection in the early stages of embryonic development [198]. Despite the clearly confirmed possibility of SARS-CoV-2 infection during the peri-implantation period, the transplacental route of viral transmission seems to be uncommon, especially in the antenatal period. It was reported that term placenta expresses low to no ACE2. Lack of this key protein for SARS-CoV-2 infection makes it difficult for the virus to pass from the maternal blood to the fetus or amniotic fluid [209]. However, in the case of vertical transmission in which the viral genome was detected in term placentas, these cases were associated with a strong maternal proinflammatory response [207, 209–211]. Local degranulation of MCs may be highly responsible for these pathologic findings [6, 13]. Thus, SARS-CoV-2 can be associated with a rare set of placental lesions that can lead to fetal demise, preterm birth, or growth restriction. The massive placental damage is indirectly induced by the virus with subsequent trophoblast necrosis and massive inflammation in the villous chamber; in a similar manner, diffuse alveolar damage is noted in adults infected by SARS-CoV-2 [210]. Again, placental MCs may represent an important source of mediators that can activate other immune cell types during SARS-CoV-2 infection, and these cells have the potential to be a major contributor to the spread of the virus in cases of vertical transmission [14, 119]. Demonstrable placental SARS-CoV-2 placentitis is an uncommon but readily recognizable complication of maternal COVID-19 with potential use as a marker of vertical transmission [211].

In contrast to term placenta, single-cell transcriptomic studies of the early placenta (6–14 weeks of gestation) revealed stable coexpression of ACE2 and TMPRSS2 in stromal and perivascular cells in the decidua and villous cytotrophoblasts and syncytiotrophoblasts. This finding may suggest that women infected early in pregnancy could potentially pass SARS-CoV-2 to the fetus through the transplacental route [207].

Interestingly, the gastrointestinal tract may also be important for the route of vertical transmission given the high coexpression of both ACE2 and TMPRSS2 in the human fetal intestine across the first and, particularly, the second trimester. Intrauterine infection through the gastrointestinal tract requires SARS-CoV-2 entry into the fetal gastrointestinal lumen through fetal swallowing of infected amniotic fluid [207]. Developing MCs were detected in the mucosa of the fetal gastrointestinal tract, but their possible role in vertical transmission was not investigated [212].

Although the course of SARS-CoV-2 infection is asymptomatic in 70-80% of cases, observed symptoms in the vast majority of pregnant woman are benign, and the risk of vertical transmission is negligible, monitoring for adverse consequences of COVID-19 during pregnancy is reasonable [213]. Both the mother and especially the child may show long-term health effects of maternal immune system activation (including MCAS) in response to SARS-CoV-2 [205, 214]. The available data do not currently demonstrate an increased teratogenic potential of SARS-CoV-2, and limited evidence has not demonstrated any association between COVID-19 and an increased risk of miscarriage [215].

## 5. Concluding Remarks

Pregnant women do not appear to be more likely to contract SARS-CoV-2 than the general population or women of childbearing age [216]. However, intensive care admission and invasive ventilation are more common among pregnant women with COVID-19 compared with infected nonpregnant women of the same age [215, 217]. Analogous to respiratory function, it cannot be excluded that the cytokine storm accompanying SARS-CoV-2 infection and MCAS during COVID-19 may indirectly affect female reproductive function, especially in more severe cases [38, 204, 205]. The demonstrated roles of MCs and MC mediators in the human female reproductive system in health and disease have been presented elsewhere (see Table 1). Neutralization of upstream histamine, a major mediator derived from MCs, inhibits the nuclear translocation of NF-*κ*B, thereby preventing the release of the proinflammatory cytokines interleukin- (IL-) 1*β*, TNF-*α*, IL-6, and IL-10 [218]. This effect has been successfully used in the MC-targeted treatment of chronic dyspareunia, vaginitis, and dysfunctional uterine bleeding [219]. Despite the fact that COVID-19 hyperinflammation and post-COVID-19 illness may be rooted in MCAS, the available clinical data do not provide grounds for treating this mechanism as a significant threat to female reproductive functions, including pregnancy [214]. Understandably, severe COVID-19 reduces female fertility and has been associated with impaired fetal growth during pregnancy, an approximate 2-fold increased risk of stillbirth, 3-fold increased risk of preterm birth (likely influenced by iatrogenic deliveries) and prematurity-related worse perinatal outcomes [215].

It should be noted that the pathophysiological spectrum of MCAS is extremely broad and heterogeneous with a strong female predilection. The above and environmental changes inducing epigenetic mechanisms may explain the increasing incidence of MCAS, which is estimated to be up to 17% [220]. Specific research in the pregnant or postpartum MCAS population related to SARS-CoV-2 infection and COVID-19 has not been performed to date. Data continue to accumulate, including those regarding the long-term influence of COVID-19 on the course of both preexisting and SARS-CoV-2-induced MCAS and female fertility. This is especially true for cases of so-called long COVID or post-acute COVID-19 when females experience lingering COVID-19 symptoms for weeks and months after this initial or acute phase of the infection [221]. It is noteworthy that long COVID with high residual cardiovascular risk and persistence of blood chemistry of inflammation and procoagulative state resembles many aspects of MCAS [222]. For example, in both pathologies, NLRP3 inflammasome (hyper)activation may be crucial in the consolidation of coagulation disorders within the female reproductive organs [223, 224]. Further studies are needed to determine whether MCAS-related clotting disorders (e.g., microcoagulopathy) are subjected to long-term modulation by SARS-CoV-2 infection with possible consequences on female reproductive function.

## Figures and Tables

**Table 1:** Proven roles of mast cells (MCs) and MC mediators in the human female reproductive system in health and disease.

MC localization	Modulated physiological or pathological process	Characteristic features: MC number/distribution, MC mediators/receptors involved	Related complication, condition, or disease	References
Ovary	Folliculogenesis, ovarian follicle selection, growth and ovulation	↑ MC number in the human ovarian medulla/stroma during the proliferative phase; ovarian MCs correspond with interleukin 8 (IL-8) staining cells; exogenous IL-8 induces a similar increase in follicular growth to that produced by the luteinizing hormone (LH) surge; MC-derived histamine may regulate the development of ovarian follicles by apoptosis	Numerous primary or undeveloped ovarian follicles, anovulation	Goto et al. 1997 [15]; Szukiewicz et al. 2007 [16]; Field et al. 2014 [17]; Szukiewicz et al. 2007 [18]
	Ovulation	MCs-derived histamine stimulates ovarian contractility, ovulation and follicular progesterone secretion; ↑ expressions of histamine H1 and H2 receptors in the preovulatory period within the growing ovarian follicles	Ovulatory disorders	Krishna et al. 1989 [19]; Szukiewicz et al. 2006 [20]
	Ovulation and luteinization	Histamine and TNF-*α* release from degranulating stores (MCs within follicular wall) during ovulation	Ovulatory disorders and/or corpus luteum dysfunction	Field et al. 2014 [17]; Galvão et al. 2018 [21]
	Altered neuroimmune communication	↓ MC number in polycystic and ↓↓↓ MC number in postmenopausal ovaries with accompanying increase in nerve fibers in the corticomedullary region; more tryptase-positive MCs than chymase-positive MCs in the interstitial cortex and the medulla of polycystic ovaries (PCO) compared to normal cyclic ovaries; nerve growth factor (NGF) production by MCs suggests an interaction between MCs and nerve fibers via high affinity NGF tyrosine kinase receptor TrkA and low affinity receptor p75NTR	Polycystic ovary syndrome (PCOS); menopause	Heider et al. 2001 [22]; Krishna et al. 2001 [23]; Chang et al. 2019 [24]
	Peritumoral MC infiltration	↑ number of activated MC promotes tumor growth and spread by the release of proangiogenic factors (e.g., VEGF), degradation of the extracellular matrix (e.g., proteases), and direct and indirect immune suppression (e.g., IL-10 and TGF-*β*1)	Ovarian cancer progression	Chan et al. 2005 [25]; Oldford and Marshall 2015 [26]; Komi and Redegeld 2020 [27]

Fallopian tube	Microcirculation of blood within fallopian tube wall	MCs regulate the microcirculatory blood stream of the fallopian tubes through release of vasoactive and angiogenic factors; volume and degree of the MC degranulation depends on the menstrual cycle phase, age and part of the fallopian tube.	Changes in the net luminal fluid secretion and absorption during the menstrual cycle	Glukhovets et al. 1980 [28]
	Foreign body inflammatory response	In contraceptive intrauterine devices (IUD) users ↑ MC number were reported both in the muscularis externa and the lamina propria of the tubal wall; most MCs of the muscularis externa were more closely related to smooth muscle cells than to blood vessels.	Increased risk of pelvic inflammatory disease and the ectopic pregnancies amongst women using IUD	Sandvei R et al. 1986 [29]

Peritoneal fluid	Endometriosis	↑ MC count with increased release of chemoattractant cytokines, such as IL-8 and the monocyte chemoattractant protein-1 (MCP-1/CCL2); severity of endometriosis is positively correlated with the increase of both IL-8 and MCP-1/CCL2 in peritoneal fluid. MCs mediators may directly suppress sperm motility; sperm interaction with the MC (LAD2) surface induces MC-degranulation in the peritoneal fluid of endometriosis patients.	Pelvic pain, endometriosis-related dysmenorrhea, growth of endometriotic foci with inflammatory response and peritoneal adhesions, infertility Endometriosis associated infertility by suppression sperm cell function including motility	Oral et al. 1996 [30]; Broi et al. 2019 [31] Menzies *et al.* 2011 [13]; Borelli et al. 2019 [32]; Broi et al. 2019 [31]

Uterus: endometrium/decidua+myometrium	Endometrial proliferation	↑ uterine MC counts in the menstrual and late secretory stages of the menstrual cycle; the pattern of MC cell ultrastructure is not associated with a particular tissue component in the uterine wall at any stage of the menstrual cycle; total degranulation of MCs is not observed during menstruation; in the late menstrual phase, degranulation by vacuolation and extensive invagination take place in the endometrial/myometrial junction and in the myometrium	Changes in functional status and secretory activity of MC within the endometrium during proliferative secretory and premenstrual stages of the menstrual cycle	Drudy et al. 1991 [33]; Sivridis et al. 2001 [34]
	Postmenopausal endometrium	↓ MC number; large numbers of MCs in the myometrial side of the endometrial/myometrial junction and in the deeper layers of the myometrium; no invagination of the MC membrane and lack of MCs with particulate granules suggest no impact on the functional layer of endometrium	Postmenopausal status of the functional layer within uterine mucous membrane	Drudy et al. 1991 [33]
	Increased endometrial proliferation	Hyperactivation of MCs; >7-fold increase in the numbers of activated MCs expressing tryptase in endometrial polyps; ↑ densities of all MC types compared to normal endometrium; ↑ numbers of chymase^+^ and c-Kit^+^ endometrial MCs	Formation of endometrial polyps and polyp-related abnormal uterine bleeding or infertility	Al-Jefout et al. 2009 [35]; El-Hamarneh et al. 2013 [36]
	Implantation	Estradiol (E_2_) and progesterone (P4)—governed migration of MCs from the periphery to the uterus and MC degranulation with the release of key factors (e.g., histamine, tryptase, VEGF, and metalloproteinases (MMPs)) during the embryo implantation in the posterior superior wall of the uterus; MC-derived histamine promoting trophoblast invasion, growth, and the expression of adhesion molecules; ↑ MC count within the entire layers of the endometrium with proinflammatory milieu in recurrent pregnancy loss (e.g., proven role of TNF-*α* excess in abortion).	Normal implantation, implantation-stage early abortion or recurrent pregnancy loss	Jensen et al. 2010 [37]; Elieh Ali Komi D et al. 2020 [38], Woidacki et al. 2014 [39]; Saito 2005 [40]; Matsuno et al. 2019 [41]
	Implantation and placenta formation	Decidual MCs show an Fc*ε*RI*α*-positive (+)Kit(+)tryptase(+)chymase(+) phenotype; a larger proportion of tryptase(+); decidual MCs of parous women express a human-specific protein killer cell Ig-like receptor (KIR) 2DL4 (CD158d), a receptor for human leukocyte antigen G (HLA-G) expressed on human trophoblasts; KIR2DL4 stimulation with agonistic antibodies and recombinant HLA-G protein may enhance establishment of pregnancy by induction of leukemia inhibitory factor (LIF) and serine proteases release from MCs, in addition to suppressing mast-cell-mediated allergic/inflammatory reactions (downregulation of the Kit-mediated and FcɛRI-mediated responses); histamine from MCs is involved in differentiation of trophoblast cells toward extravillous, endovascular trophoblasts by stimulation of integrin *α*V-*β*3 (a vitronectin receptor) expression on trophoblast cells	Establishing pregnancy via killer cell Ig-like Receptor (KIR2DL4) expressed on the surface of decidual MCs; invasion of the uterine wall and the spiral arteries by extravillous trophoblasts (interstitial and endovascular invasions, respectively)	Derbala et al. 2018 [42]; Ueshima et al. 2018 [43]; Kataoka et al. 2020 [44]; Szewczyk et al. 2005 [45]
	Foreign body inflammatory response	↑ density of MCs in the endometrium of contraceptive intrauterine devices (IUD) users after 3 to 24 months' use of IUD, independent of IUD type	Changed inflammatory cytokine profiles of endometrium at peri-implantation period corresponding to a contraceptive effect of IUD	Yin et al. 1993 [46]; Chou et al. 2015 [47]
	Contractile activity	↑ density of MCs in pregnant vs. non-pregnant myometrium; tryptase(+)/chymase(+) MCs are predominant in nonpregnant myometrium, whereas tryptase(+) MC dominate in pregnant myometrium; histamine H_1_ receptor antagonists partially inhibit uterine contractions; uterine MC degranulation, or the effects of their mediators, modulates contractility of pregnant uterus	Term or preterm uterine contractions including abortion and preterm/term delivery	Garfield et al. 2006 [48]; Bytautiene et al. 2004 [49]; Szukiewicz et al. 1995 [50]
	Endothelin-1 (ET-1) production in the myometrium	MC-derived chymase is included in the chymase–ET-1 system operating in the myometrium during pregnancy; the number of MCs and production of ET-1 are significantly higher in myometrium from patients with severe preeclampsia compared to those from normal pregnant women	Chymase-dependent production of ET-1 in the myometrium during normal pregnancy and preeclampsia	Mitani et al. 2002 [51]; D'Orléans-Juste et al. 2008 [52]
	Cervical ripening	Increased influx of MCs to the cervix during pregnancy; physiological or pathological stimulation of the secretory activity of cervical MCs may lead to the local increase in a number of MC-specific proteases, including the neutral proteases chymase, tryptase, and carboxypeptidase A.	Cervical tissue remodeling: in first trimester symptomatic miscarriage, in term/preterm pregnancy (contributing to term/preterm delivery), and in the postpartum period.	Elieh et al. 2020 [38]; Norström et al. 2019 [53]; Saito 2005 [40]

Placenta	Angiogenesis	Placental MCs are an important source of potent proangiogenic factors (e.g., histamine, VEGF, bFGF, TGF-beta, TNF-alpha, and IL-8) and a source of extracellular matrix-degrading proteinases; changes in both the number of MCs and angiogenesis mediator concentrations were reported in placental tissue pathologies including diabetes and fetal growth restriction (FGR); ↑ placental expression of histamine H_4_ receptors in diabetes may enhance MC chemotaxis towards angiogenic sites.	Normal placental vascularization; defective and incomplete placental vascularization in IUGR; hypervascularization in diabetes mellitus	Kurihara-Shimomura et al. 2020 [54]; de Souza Junior et al. 2015 [55]; Szukiewicz et al. 1999 [56]; Szukiewicz et al. 2003 [57]; Szewczyk et al. [58]
	Trophoblast invasion and spiral artery remodeling	MCs are important for proper development of the placental bed; ↑ chymase expression and activity in placental trophoblasts and in the maternal vascular endothelium in pregnancy induced hypertension; differential MC distribution and corresponding changes in the concentration of histamine are involved in the defective placental vascularization in preeclamptic placentas; both ↑ and ↓ MC numbers were reported in the villous part of the placenta in preeclampsia; unlike in normal placentae, in preeclamptic placentae histamine does not stimulate expression of integrin *α*v-*β*3 which is the necessary integrin to ensure trophoblast invasiveness.	Normal placental vascular bed formation; pregnancy-induced hypertension including the most prevalent hypertensive disorders of pregnancy: preeclampsia and eclampsia; FGR caused by insufficient remodeling of spiral arteries	Faas and De Vos 2018 [59]; Wang and Alexander 2013 [60]; Szewczyk et al. 2012 [61]; Szukiewicz et al. 1999 [62]; Mitani et al. 2002 [51]; Szewczyk et al. 2008 [63]; Meyer et al. 2017 [64]
	Apoptosis	MC-derived histamine inhibits the apoptotic activity in trophoblast cells via histamine H_1_ receptor and further influences the process of trophoblast invasion and differentiation.	Placental apoptosis and related differentiation of the trophoblast and placental turnover	Wu et al. 2012 [65]; Pyzlak et al. 2010 [66]; Szewczyk et al. 2005 [45]; Liu et al. 2004 [67]
	Contractile activity of the uterus including initiation of labor	↑ density of MCs near the fetal surface of the placenta and in connective tissue foci; contractile activity of the uterus during normal vaginal delivery decreases histamine concentration in the placental tissue near the maternal surface of the placenta; MC distribution in placental tissue and membranes as well the degree of their secretory activation influence contractile activity of the uterus in health and disease.	Normal initiation of labor at term and premature birth with preterm onset of the contractile activity of the uterus	Szukiewicz et al. 1995 [50]; Needham et al. 2016 [68]
	Allergic reaction within the human placenta	↑ MC number and/or increased level of MC activation result in high levels of MC mediators in placental tissue in allergic mothers; allergen-induced placental cytokine and chemokine release include histamine, CXCL10, CXCL11, CCL17, CCL22, IL-6, and TNF.	Allergens induce placental cytokines and chemokines distinctly in allergic and healthy mothers influencing the prenatal development of the immune system; increased rate of immune disorders in childhood including allergies should may occur	Abelius et al. 2014 [69]; Mikkelsen et al. 1994 [70]; Straubinger et al. 2014 [71], Papadogiannakis et al. 2019 [72]

Endometrial tissue outside the uterine cavity	Pain induction and mediation	↑ MC number and ↑ number of degranulating MCs in endometriotic foci compared to nonaffected tissues; cross-talk between MCs and neurons is responsible for pain mediation; MCs may contribute to the development of pain and hyperalgesia in endometriosis, possibly by a direct effect on nerve structures; ↑ E_2_ concentrations may be a key factor for degranulation and recruitment of MCs in ovarian endometriomas with a key role in endometriosis-associated dysmenorrhea.	Endometriosis-related chronic and neuropathic pain including dysmenorrhea	Anaf et al. 2006 [73]; Zhu et al. 2019 [74]; D'Cruz et al. 2007 [75]; Zhu et al. 2018 [76]
	Fibrosis and fibrotic scarring	Invasion of MCs, degranulation, and proliferation of interstitial component are observed in endometriotic lesions; the Janus kinase 3 (JAK3) is abundantly expressed in MCs and is required for the full expression of high-affinity IgE receptor-mediated MC inflammatory sequelae including fibrosis and increased risk of adhesion development; ↑ numbers of activated MCs in endometriosis are strongly positive for corticotropin-releasing hormone (CRH) and urocortin (Ucn)—the peptides activating MCs and contributing to the fibrosis and inflammation.	Fibrous adhesions in endometriotic lesions	Anaf et al. 2006 [73]; Kirchhoff et al. 2012 [77]; Kempuraj et al. 2004 [78]
	Angiogenesis	Activation of MCs in situ causes local MC-mediated angiogenesis; C-C Motif Chemokine 8 (CCL8) promotes both in vitro and in vivo angiogenesis via the CC chemokine receptor 1 (CCR1); ↑ CCL8 in MCs was reported in the coculture with endometrial cells; ectopic endometrium and the serum of patients with endometriosis revealed ↑ CCL8 expressions; ↑ CCR1 perivascular expression was reported in the ectopic endometrium in ovarian endometriomas.	Ectopic angiogenesis in endometriotic foci	Norrby 1995 [79]; Xue et al. 2020 [80]; Li et al. 2020 [81]; Binda et al. 2017 [82]

**Table 2:** Main categories of human mast cell- (MC-) derived mediators and their well-known topical effects.

Mediator category	Selected overall profile of the local activities	References
*Preformed in MC secretory granules*		
Proteases: Tryptases Chymases Carboxypeptidase A Cathepsin G Renin Granzyme B	Neutral proteases play dual roles in inflammatory states depending on immunologic context, exerting proinflammatory and protective effects by activation/inhibition of the multiple respective cytokines within the signaling pathways; these activities result from proteolytic properties including angiotensin converting enzyme (ACE) activity, broad-spectrum antibacterial action against Gram-negative and Gram-positive bacteria; extracellular matrix (ECM) breakdown at inflammatory sites, ability to degrade some neuropeptides and toxins (neurotoxins), cleavage of receptors, platelet activation, and induction of airway submucosal gland secretion	Galli et al. 2020 [92]; Silver et al. 2004 [93]; Maaninka et al. 2018 [94]; Heutinck et al. 2010 [95]; Peljer et al. 2007 [96]; Peljer et al. 2010 [97]; Caughey 2016 [98]; Wang et al. 2014 [99]
Biogenic amines:		
Histamine	Histamine action is mediated by histamine receptors H1, H2, H3, and H4 expressed in target cells; typical effects of histamine release include increased venular permeability with cutaneous flushing (H1, H2), increased heart rate and cardiac output (H1), bronchoconstriction (H1), increased mucus production in airways, nasal (H1) or generalized (H2), increased gastric acid secretion (H2), positive chemotaxis of neutrophils, T cells and eosinophils (H1) or neutrophil and eosinophil influx inhibition (H2), autoregulation of histamine release in brain (presynaptic H3), modulation of T helper type 2 (Th2) cell responses (H4)	Tiligada and Ennis 2020 [100]; Walter & Stark 2012 [101]; Thangham et al. 2018 [102]; Hattori et al. 2017 [103]
Serotonin (5-HT)	Serotonin is produced in human MCs during fetal development (e.g., placental MCs) and, to a lesser degree, in adult life; mastocytosis may induce 5-HT production in MCs; 5-HT signaling affects fetal brain development and placenta-derived 5-HT may be important for normal fetal brain development; 5-HT from MCs contributes to behavioral and physiological functions of the hippocampus	Bonnin and Levitt 2011 [104]; Ranzil et al. 2019 [105]; Kushnir-Sukhov et al. 2007 [106]; Ritter et al. 2012 [107]; Nautiyal et al. 2012 [108]
Proteoglycans:		
Heparin Heparin sulfate Chondroitine sulfate Dermatan sulfate Serglycin proteoglycan	The proteoglycan serglycin carries an array of glycosaminoglycan side chains, sometimes heparin, sometimes chondroitin or dermatan sulphate. The members of proteoglycan family play essential role in regulation of MC granule storage and the release of secretory granule compounds, having an impact on the fate of the respective compounds after degranulation, affect the enzymatic properties of MC proteases and may promote apoptosis. Following MC degranulation heparin is probably involved in regulation of interstitial clotting but not in clotting in the vessels	Rönnberg et al. 2012 [109]; Mulloy et al. 2017 [110]; Zehnder and Galli 1999 [111]

*De novo synthesized lipid mediators derived from arachidonic acid (eicosanoids)* Prostaglandins (PGs) Leukotrienes (LTs) Platelet activating factor (PAF)	At a tissue level, PGD_2_, the major PG produced by activated MCs and cysteinyl LTs (LTC_4_ and its active metabolites LTD_4_ and LTE_4_, as well as far lesser amounts of LTB_4_) produce increased vascular permeability, inflammation, pain, bronchoconstriction, increased uterine activity and vasoconstriction or vasodilation; PAF possesses high chemoattractant activity to neutrophils, eosinophils, monocytes, and macrophages and stimulates cytokine production by macrophages;	Sahid and Kiyoi 2020 [112]; Liu et al. 2017 [113]; Nakamura and Murata 2018 [114]; Theoharides et al. 2012 [115]

*Cytokines including chemokines (selected)* Interleukins: IL-1, IL-3 ,IL-4, IL-5, IL-8,IL-9, IL-10, IL-31, IL-33 Growth factors: TNF-*α*, TGF-*β*1, TGF-*β*2, NGF, SCF, PDGF, VEGF, GM-CSF Chemokines: CCL2, CXCL8 (IL-8), CX3CL1,	MCs release multifunctional cytokines involved in recruitment and activation of other cells participating in immune and inflammatory response; the cytokine profile heterogeneity reflects the differences in the secretory granule protease phenotypes between MCs and the tissue localization; depending on the stimulus, MCs calibrate their pattern of cytokine release, and as the immunoregulatory cells, can alter their response ranging from pro-inflammatory to anti-inflammatory	Elieh Ali Komi et al. 2020 [116]; Frossi et al. 2018 [9]; Moon et al. 2014 [117]

## Data Availability

Data availability issue does not apply to this review paper. All data are from the published papers and are included in the references.

## Abbreviations

5-HT: 5-Hydroxytryptamine, serotonin
*α*V-*β*3: Member of the integrin family, also known as a vitronectin receptor
ACE: Angiotensin-converting enzyme
ACE2: Angiotensin-converting enzyme 2 receptor
Akt: Protein kinase B
AMH: Anti-Mullerian hormone
ANPEP: Cell membrane alanyl aminopeptidase
ART: Assisted reproductive technology
bFGF: Basic fibroblast growth factor
CCL2: C-C motif chemokine ligand 2, monocyte chemoattractant protein-1 (MCP-1)
CCL8: C-C motif chemokine 8
CCL17: C-C motif chemokine ligand 17
CCL22: C-C motif chemokine ligand 22
CCR1: C-C chemokine receptor type 1
CD117: The c-kit protooncogene product
CLEC4G: C-type lectin domain family 4 member G
CLR: C-type lectin receptors
COVID-19: Coronavirus Disease 2019
CRH: Corticotropin-releasing hormone
CTSL: Lysosomal cysteine peptidase cathepsin L
CTSB: Cathepsin B
CX3CL1: C-X3-C motif chemokine ligand 1 or fractalkine
CXCL8: C-X-C motif chemokine ligand 8 or interleukin 8 (IL-8)
CXCL10: C-X-C motif chemokine ligand 10, also known as interferon-gamma-induced protein 10 (IP-10)
CXCL11: C-X-C motif chemokine ligand 11
DAG: Diacylglycerol
E2: Estradiol
ECM: Extracellular matrix
EGFR: Epidermal growth factor receptor
ER*α*, ER*β*: Estrogen receptors alpha, beta
ET-1: Endothelin 1
Fc*ε*RI*α*: High-affinity IgE receptor
FGR: Fetal growth restriction
FSH: Follicle-stimulating hormone
GM-CSF: Granulocyte-macrophage colony-stimulating factor
GPER: G protein-coupled estrogen receptor
GRP78: Glucose regulated protein 78
H1, H2, H3, H4: Histamine receptors: H1 H2, H3 and H4, respectively
HLA-G: Human leukocyte antigen
IFITM1: Interferon-induced transmembrane protein 1
IgE: E class immunoglobulin
IL-1, IL-3, IL-4, IL-5, IL-6, IL-8, IL-9, IL-10, IL-31, IL-33: Interleukins
IP3: Inositol-1,4,5-triphosphate
ITAMs: Immunoreceptor tyrosine-based activation motifs
IUD: Contraceptive intrauterine device
IVF: In vitro fertilization
JAK3: Janus kinase 3 of the killer cell Ig-like receptor (KIR)
LAD2: Laboratory of allergic diseases 2 human MCs
LAT1, LAT2: Linkers for activation of T cells family, member 1 and 2, respectively
LH: Luteinizing hormone
LIF: Leukemia inhibitory factor
LTs (LTC4, LTD4, LTE4, LTB4): Leukotrienes: LTC4, LTD4, LTE4, LTB4
MAPK: Mitogen-activated protein kinases
MC, MCs: Mast cell, mast cells
MCAS: Mast cell activation syndrome
MCP-1/CCL2: Monocyte chemoattractant protein-1
MCT: Tryptase+ but chymase– MC subtype
MCTC: Tryptase+ and chymase+ MC subtype
miRNAs (e.g., miR-142-3p, miR-126, miR-155, and miR-221/222): MicroRNAs
MRGPRX2: Mas-related G-protein coupled receptor member X2
MMPs: Metalloproteinases
mTOR: Mammalian target of rapamycin (mTOR)
nc-MCAS: Nonclonal MC activation syndrome
NGF: Nerve growth factor
NLRP3: Inflammasome (NOD-, LRR-, and pyrin domain-containing protein 3)
NRP1: Neuropilin-1
NF-*κ*B: Nuclear factor kappa-light-chain-enhancer of activated B cells
P4: Progesterone
p75NTR: Low-affinity receptor for neurotrophins
PAF: Platelet-activating factor
PAMPs: Pathogen-associated molecular patterns
PCO: Polycystic ovaries
PDGF: Platelet-derived growth factor
PGs: Prostaglandins
PIP3: Phosphatidylinositol (3,4,5)-trisphosphate
PR-A, PR-B: progesterone (P4) receptors A, B
ROS: Reactive oxygen species
SARS-CoV-2: Severe acute respiratory syndrome coronavirus 2
SCF: Stem cell factor
scRNAseq: Single-cell RNA-sequencing
GF-*β*1, TGF-*β*2: Transforming growth factors: *β*1 and *β*2, respectively
Th2 cell: T helper type 2 cell
TLRs: Toll-like receptor
TMPRSS2, TMPRSS4, TMPRSS11A: Transmembrane serine proteases: 2, 4 and 11A, respectively
TNF-*α*: Tumor necrosis factor *α*
TrkA: Neurotrophic tyrosine kinase receptor (NTRK1)
TSH: Thyroid-stimulating hormone
Ucn: Urocortin
VEGF: Vascular endothelial growth factor
Zeb2: Zinc finger E-box-binding homeobox 2 or Smad interacting protein 1 (Sip1)
